# Tag-mediated single-step purification and immobilization of recombinant proteins toward protein-engineered advanced materials

**DOI:** 10.1016/j.jare.2021.06.010

**Published:** 2021-06-15

**Authors:** Ana I. Freitas, Lucília Domingues, Tatiana Q. Aguiar

**Affiliations:** CEB - Centre of Biological Engineering, University of Minho, 4710-057 Braga, Portugal

**Keywords:** Protein tags, Solid-binding peptides, Non-covalent binding, Covalent binding, Protein-engineered materials, Protein purification/immobilization

## Abstract

**Background:**

The potential applications of protein-engineered functional materials are so wide and exciting that the interest in these eco-friendly advanced materials will further expand in the future. Tag-mediated protein purification/immobilization technologies have emerged as green and cost-effective approaches for the fabrication of such materials. Strategies that combine the purification and immobilization of recombinant proteins/peptides onto/into natural, synthetic or hybrid materials in a single-step are arising and attracting increasing interest.

**Aim of Review:**

This review highlights the most significant advances of the last 5 years within the scope of tag-mediated protein purification/immobilization and elucidates their contributions for the development of efficient single-step purification and immobilization strategies. Recent progresses in the field of protein-engineered materials created using innovative protein-tag combinations and future opportunities created by these new technologies are also summarized and identified herein.

**Key Scientific Concepts of Review:**

Protein purification/immobilization tags present a remarkable ability to establish specific non-covalent/covalent interactions between solid materials and biological elements, which prompted the creation of tailor-made and advanced functional materials, and of next-generation hybrid materials. Affinity tags can bind to a wide range of materials (of synthetic, natural or hybrid nature), being most suitable for protein purification. Covalently binding tags are most suitable for long-term protein immobilization, but can only bind naturally to protein-based materials. Hybrid affinity-covalently binding tags have allowed efficient one-step purification and immobilization of proteins onto different materials, as well as the development of innovative protein-engineered materials. Self-aggregating tags have been particularly useful in combination with other tags for generating protein-engineered materials with self-assembling, flexible and/or responsive properties. While these tags have been mainly explored for independent protein purification, immobilization or functionalization purposes, efficient strategies that combine tag-mediated purification and immobilization/functionalization in a single-step will be essential to guarantee the sustainable manufacturing of advanced protein-engineered materials.

## Highlights

• Recent advances in tag-mediated protein purification/immobilization are highlited.
• Their contribution for single-step protein purification & immobilization is clarified.
• Innovative protein-engineered materials developed using protein tags are outlined.
• The impact of these technologies on the fabrication of hybrid materials is discussed.
• Future opportunities created by these new technologies are identified.

## Graphical abstract

**Figure:**
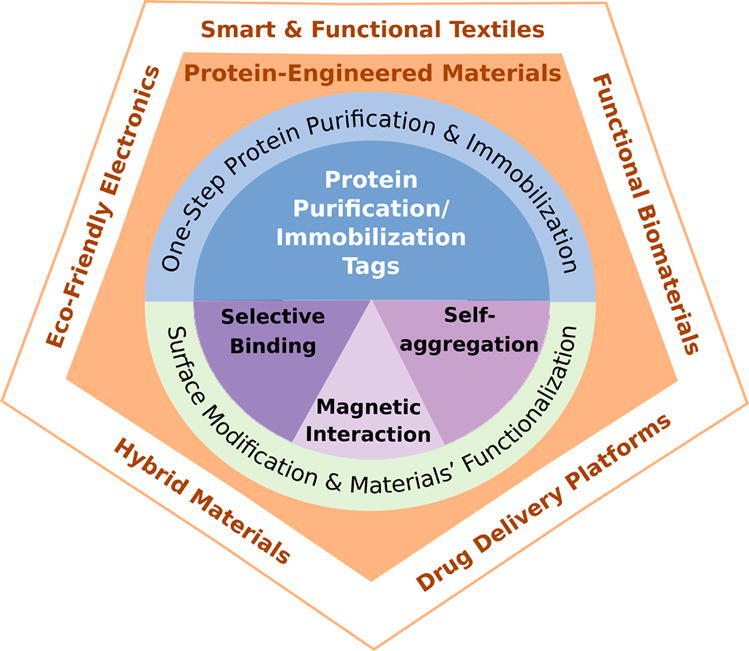

## Introduction

The increasing demand for biocompatible, bioactive and/or environmentally friendly materials that can be easily fabricated and tuned, has joined scientists from the material science and genetic engineering fields in the effort to develop bioinspired engineered materials combining several of the advantages of natural and synthetic materials [1], [2], [3]. In this context, protein-engineered materials, which span from entirely natural protein-based polymers to hybrid materials comprising inorganic/synthetic materials functionalized with peptides/proteins, capture the best of both worlds (i.e., biofunctionality and customizability) [1], [4], [5].

By using genetic engineering methods and recombinant DNA technology, synthetic genes encoding precisely designed amino acid sequences can be constructed and inserted into host organisms able to produce the desired peptides/proteins in a recombinant form [6]. Recombinant proteins can then be purified to be used in key research and/or industrial applications in the areas of biotechnology (e.g., biosensing, biocatalysis and bioremediation) [7], [8], biomedicine (e.g., drug delivery, vaccine development and biomedical implants) [9], [10] and food [11], textile [12] and paper industry [13]. The advent of this technology made available a wide variety of native-based proteins at a large scale, genetic variants of existing proteins and entirely new proteins engineered with fine-tuned properties [6].

Several prokaryotic (e.g., *Escherichia coli* and *Bacillus subtilis*) and eukaryotic hosts (e.g., yeast and animals’ cells) have been engineered for the production of recombinant proteins, to reduce the complexity of the bioprocess and to improve the product quantity and quality. All have advantages and limitations and therefore should be selected according to the characteristics of the protein to be expressed [6], [14]. For instance, in *E. coli*, purification is more complex because recombinant proteins are mainly produced inside the cells, while in the yeast expression system *Pichia pastoris* production is typically directed into the extracellular medium, where there are less contaminants [15]. On the other hand, the *E. coli* expression system is highly efficient in incorporating foreign DNA and in expressing recombinant proteins at very high rate and yield [16]. Therefore, whenever possible, *E. coli* is the heterologous host of choice given its easy manipulation, fast-growing and cost-effectiveness [14], [16]. So, continuous efforts have been made to overcome some of the limitations of this expression system.

One of the major advances in this field was the discovery of fusion partners (or simply “tags”) which can be either a protein domain, a small peptide or even an enzyme fused at either the N- or C-terminus of the target protein to enhance its expression, solubility (i.e., correct folding) and/or purification [16], [17]. The use of fusion tags is nowadays one of the most powerful strategies for obtaining recombinant proteins on a large scale with high yield and purity, independently of the host expression system used [15]. Another major upgrading in this field was the discovery of virus proteases for targeted tag removal. While some tags do not need to be removed from the sequence of the protein of interest, others negatively impact the biological activity of the protein of interest and thus their removal stands as a prerequisite [15], [18]. Therefore, a good fusion tag should display the following properties: (i) versatility (e.g., able to be fused with a wide range of target recombinant proteins), (ii) non-interference with the correct folding of the target protein, thus allowing biological active conformation; and (ii) easy release from the target protein either by chemical or enzymatic cleavage methods, if needed [14], [18].

In the past decades, several fusion tags have been developed for recombinant protein production, comprising multiple purposes and applications [17], [18]. While some tags provide solubility enhancement of the target protein, others are highly efficient for detecting and purifying recombinant fusion proteins from crude biological extracts in a single step, following selective binding to a range of materials that display specific ligands on their surfaces – the so-called affinity tags [17], [18]. Due to their unique features, affinity tag-based separation and recovery systems are nowadays indispensable tools for the massive production, identification and purification of recombinant proteins/peptides to be used in different applications [18], [19], [20]. For instance, in biomedicine this technology boosted the design and production of antimicrobial peptides [19]. More recently, affinity tags have been also fairly used to mediate the site-directed immobilization of enzymes onto different matrices with minor effects on the biologic activity of the enzyme, as well as in the functionalization (i.e., surface modification) of a wide range of natural and synthetic materials [21], [22], [23].

Surface modification aiming at enhancing materials with biological/biomimetic properties holds the promise to fit innumerable upcoming applications ranging from regenerative medicine to smart and functional textiles, while encompassing several economic and ecological benefits [12], [24]. Moreover, the employment of recombinant proteins/peptides with new and/or enhanced functionality toward tailor-made materials with novel/improved biological and/or structural properties has been an emergent field and has opened a myriad of new perspectives in material and life sciences [24]. Materials’ functionalization can be achieved via chemical and physical techniques, such as adsorption, covalent attachment (i.e., cross-linking) and entrapment/encapsulation. Conventional chemical conjugation and covalent binding methods have been the technologies of choice to link proteins/peptides to one another or to materials, but there are some drawbacks associated to the use of such techniques [25], [26]: (i) some of the chemicals used are toxic to the environment and to biological systems (e.g., glutaraldehyde and cyanogen bromide); (ii) some of the reagents or conditions used reduce the stability/activity of proteins (e.g., organic solvents and high temperatures); (iii) in most of these techniques (e.g., glutaraldehyde-, carbodiimide-, thiol- and silane-mediated coupling methodologies) the orientation and conformation of proteins is hard to control, which may negatively impact their stability/activity. Therefore, “greener” and more efficient methodologies (e.g., which avoid or require a more reduced use of hazardous consumables) are being continuously pursued and systematically improved to ensure the sustainability and biocompatibility of functionalized materials [25], [26], [27].

Another field with great interest in new and “greener” methodologies is the field of industrial biocatalysis [28]. When compared to soluble enzymes, immobilized enzymes offer enhanced stability and easy removal from the reaction mixture, enabling repetitive use in batch and continuous bioprocesses, rapid termination of reactions, controlled product formation and the provision of flexibility to industrial bioprocesses (e.g., biofuel production), thus improving their cost-effectiveness, while decreasing their ecological impact [28]. Therefore, for applied, ecological and economic reasons, research focusing on integrating recombinant protein purification and immobilization in a single step is simultaneously attracting the attention of several industrial fields, as such approaches hold the potential to simplify the fabrication process of protein-engineered materials in an eco- and economically sustainable way.

Most of the integrated strategies developed for single-step protein purification and immobilization use protein tags to mediate the specific linkage of the protein of interest to a desired matrix [29], [30], [31], [32]. Since these tags have been mainly explored for independent protein purification, immobilization or functionalization purposes, this review starts by highlighting the major advances of the last lustrum concerning tag-mediated recombinant protein purification/immobilization and then offers insights into how these advances have been contributing for the development of novel and efficient single-step purification and immobilization strategies. Given the increasing significance of tag-mediated single-step protein purification and immobilization for the material science field, this review also provides an update on the progress taking place in the field of protein-engineered materials developed using innovative protein-tag combinations, and identifies future prospects enabled by the technologies reviewed herein.

## Protein purification/immobilization tags

The development of fast, reliable and cost-effective technologies for the separation and purification of recombinant proteins has been an important prerequisite, namely in the biotechnology and biomedicine fields, where the obtainment of recombinant proteins with high yield, purity and activity is indispensable for different and multiple downstream applications [15].

Affinity protein purification using antibody-based separation or a matrix with specific ligands for affinity tag binding are commonly used approaches. In this field, various peptides have shown promise as affinity purification tags, as they exhibit low toxicity to the host cells and are shown to be more chemically stable than larger (protein) tags [18]. Some are already largely applied in single-step purification strategies, with the major advantage of being adaptable from laboratory to industrial scale in a cost-effective manner without compromising yields of purified proteins [33]. Affinity tag-based separation and recovery systems have boosted the purification of recombinant proteins, but like other protein purification systems they are not always straightforward [15]. These systems can be time-consuming, as they demand several analytical steps, and may encompass high economic and ecological costs associated with the requirement of specialized consumables and resources (namely qualified Human resources), and to the use of hazardous/pollutant reagents during the operation, respectively [15]. In response to some of these drawbacks, several tags have been screened or engineered to enable efficient and cost-effective protein purification/immobilization. For well-established tags, novel uses have also arisen, namely in single-step protein purification and immobilization. Therefore, engineered and non-engineered protein purification/immobilization tags that emerged or had novel reported uses in the last lustrum are reviewed in this section (Table 1).

**Table 1:** Protein purification/immobilization tags that emerged or had novel reported uses in the last 5 years.

Tag designation	Number of residues	Sequence	Molecular weight (kDa)	Tag location	Reference
ABD(KC)^a^	134	Large peptide/protein	14,5	C-	[23]
Alfa-tag	15	SRLEEELRRRLTE	1.7	Both	[76]
Arg-based tags	6 or 8	(R)6 or R(8)	1.0 to 1.3	Both	[48]
Car9^a^	12	DSARGFKKPGKR	1.4	Both	[50], [51]
CBMs^a^	93 to 191	Large peptide/protein	11 to 49.5	Both	[33], [60], [69]
CL7	132	Large peptide/protein	16	Both	[71]
CspB50^a^	50	QETNPTFNINNGFNDADGSTIQPVEPVNHTEETLRDLTDSTGAYLEEFQY	5.4	C-	[105]
DBD^a^	130	Large peptide/protein	14	C-	[72]
ELP	20 to 330	Large peptide/protein	2.1 to 41	Both	[99]
FLAG-tag®	8	DYKDDDDK	1	C-	[77]
Glu6^a^	6	(E)6	0.8	N-	[45]
HaloTag®	312	Large peptide/protein	34	N-	[78], [81]
HB-tag	32	ASKAQKAQAKQWKQAQKAQKAQAKQAKQAKQW	3.6	N-	[74], [75]
(HE)7^a^	7	(HE)7	1.9	N-	[46]
His-tag^a^	2 to 10	(H)2–10	0.3 to 1.4	Both	[39]
LCIa)	47	AIKLVQSPNGNFAASFVLDGTKWIFKSKYYDSSKGYWVGIYEVWDRK	5.5	Both	[132]
Lys6^a^	6	(K)6	0.8	Both	[131]
MagR^a^	130	Large peptide/protein	14.1 to 14.6	N-	[97]
MhPA14^a^	214	Large peptide/protein	22.5	Both	[70]
NCTR25^a^	25	MDHSHHMGMSYMDSNSTMQPSHHHP	2.9	N-	[47]
pSN6^a^	70 or 91	(VKTQATSREEPPRLPSKHRPG)3–4(VKTQTAS)	7.8 or 10.1	N-	[56], [57]
Protein A	252	Large peptide/protein	28.3	Both	[91], [126]
Q-based tags	6 or 7	MLAQGS or YAHQAHY	0.6 or 0.9	Both	[93], [94]
R5^a^	19	SSKKSGSYSGSKGSKRRIL	2	Both	[54]
SB7^a^	7	RQSSRGR	1	Both	[53]
SBP-tag	38	MDEKTTGWRGGHVVEGLAGELEQLRARLEHHPQGQREP or A18C variant	4.3	C-	[23], [72], [73]
SnoopCatcher	111	Large peptide/protein	12	Both	[84], [89]
SnoopTag	12	KLGDIEFIKVNK	1.4	Both	[84]
SpyCatcher	138	Large peptide/protein	15	Both	[84], [89]
SpyTag	13	AHIVMVDAYKPTK	1.5	Both	[84]
SpyTag002	14	VPTIVMVDAYKRYK	1.7	Both	[88]
SpyTag003	16	VPTIVMVDAYKRYK	1.9	N-	[20]
Trp-based tags	6	(NW)3 or (WF)3	0.9 or 1.0	N-	[49]

**Legend:** ABD, agarose-binding domain; CBM, carbohydrate-binding module; CspB, cell surface protein B; DBD, dextrose-binding domain; ELP, elastin-like polypeptide; HB, heparin-binding peptide; HE, modified His-glutamate tag; MagR, magnetoreceptor; MhPA14, marinobacter hydrocarbonoclasticus PA14 tag; LCI, liquid chromatography peak I (polymer-binding peptide); RLP, resilin-like polypeptide; SBP, streptavidin-binding peptide; *, identifies those categorized as solid-binding peptides.

a Solid-binding peptides.

**Fig. 1:**
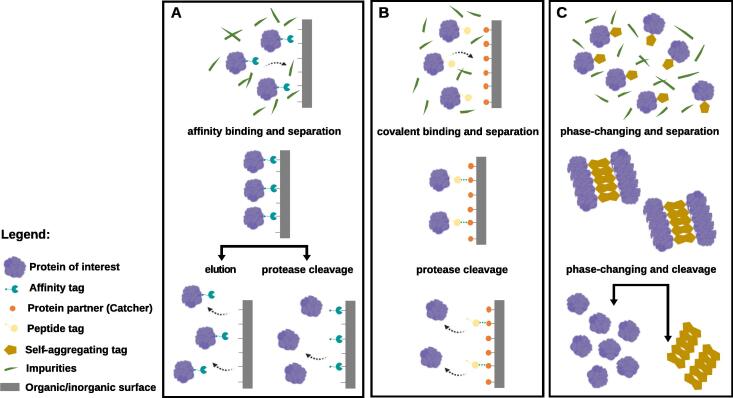
Schematic representation of the different types of protein binding toward one-step purification of recombinant proteins. (A) Classic affinity-based purification based on the non-covalent interaction between the affinity tag fused to the target protein and its specific ligand (surface); (B) Covalent-based purification based on the Tag/Catcher system, which makes use of the spontaneous isopeptide bond formation between the Catcher attached to a surface and the peptide tag fused to the target protein; (C) Column-free system for protein separation based on fusion tags with the ability to aggregate under specific environmental conditions. The formed aggregates precipitate and can be separated from impurities (crude biological extracts and/or cell lysates). After separation from impurities, the purified protein may stay immobilized onto the surface (A and B) or aggregated (C), or may be recovered by elution (A) or by separation from the tag via protease cleavage, which specifically recognizes an amino acid sequence located between the target protein and the tag (A-C).

The strength and specificity of the attractive interaction between a tag and its ligand (of biological or synthetic origin) under specific conditions is what results in a highly efficient single-step purification/immobilization of the target protein/peptide. This interaction can be either reversible (i.e., transient) or irreversible (i.e., thermodynamically very stable). Non-covalent interactions are reversible, whereas covalent interactions are usually irreversible. An exception is the coordinate covalent interaction between the His-tag and a variety of metal ions (e.g., Ni^2+^, Co^2+^, Zn^2+^, Fe^2+^), which is reversible [32]. The strength of reversible tag-ligand interactions is typically measured and reported by the equilibrium dissociation constant (K_D_) [34]. The lower the K_D_ value, the greater the affinity of the tag for its binding partner. The higher the K_D_ value, the more weakly the tag and its ligand are attracted to and bind to one another [34]. High-affinity protein tags have K_D_ values that typically lie in the micromolar to sub-micromolar range. Most of the protein affinity tags interact with their binding partners via a range of non-covalent contacts, which often dissociate under challenging conditions. Conversely, covalently binding tags establish with their ligand stable covalent interactions, providing many opportunities in a diverse range of applications such as hydrogels’ and vaccines’ development, and enzyme immobilization [35], [36]. Therefore, while most affinity tags promote reversible and non-covalent binding, most covalent immobilization tags promote irreversible binding (Fig. 1A-B). Both have been explored in single-step purification and/or immobilization of proteins and are reviewed in the sub-sections ‘Affinity tags’ and ‘Covalently binding tags’. Among these, solid-binding peptides (SBPs; also called material-binding peptides) have been the focus of great interest in recent years (Table 1) [22], [25]. SBPs exhibit selectivity (i.e., bind more preferably to the highly specific binding partner than to less specific partners) and bind with high affinity (K_D_ values ranging from 10^−6^–10^−10^ M) to a numerous range of materials such as metals and metal-oxides, semiconductors, carbon-based materials or even polymers, via non-covalent interactions [24], [25]. The binding can occur under several environmental conditions and without the need of an additional chemical reaction [25]. Moreover, SBP-mediated linkage confers directionality and orientation to the immobilized proteins/peptides without affecting their functionality [25]. Therefore, they appear as “green” and efficient alternatives to conventional chemical immobilization methods, while allowing the purification and immobilization of proteins onto natural or synthetic materials in a single step. Covalent immobilization tags such as the Tag/Catcher system recently developed (Table 1) are also gaining increasing interest, as they allow the spontaneous crosslinking of tagged proteins/peptides under physiological conditions, their oriented immobilization and the design of complex protein architectures [36], [37]. They also allow the purification and immobilization of proteins in a single step, being very versatile and particularly useful for the generation of innovative protein-based polymers [35], [37], [38].

Non-chromatographic and matrix-free systems involving the use of recently discovered magnetic or self-aggregating tags (Table 1) are also reviewed in sub-section ‘Alternative purification tags’. While magnetic tag-fusions are dependent on a magnetic field to be recovered from crude biologic extracts, self-aggregating tags form an aggregate with the protein of interest easily recovered by precipitation (Fig. 1C) and have been extensively explored for the generation of responsive materials.

### Affinity tags

**Fig. 2:**
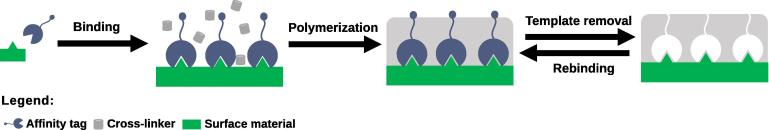
Representation of the general process of non-covalent molecular imprinting technology to create functional biosynthetic polymers via conjugation of natural and synthetic moieties. A template (affinity tag) is assembled by non-covalent binding to a surface and the polymerization is carried out around the pre-complex. The template complex is further removed from the developed biopolymer and the generated three-dimensional imprinted cavities are then exposed for further site-specific recognition (rebinding). Only tagged proteins with compatible binding sites with high selectivity can be entrapped onto the newly imprinted biopolymer, improving either purification or immobilization selectivity [21], [40], [77]**.**

Poly-His-tagging is still the most widespread and versatile strategy used to purify recombinant proteins. The His-tag is a small tag (Table 1), inexpensive to use, has low immunogenicity and minimal or no effect on the biological structure of the target protein. This tag has been for long considered a useful partner for single-step purification methods and was recently found to display affinity to new matrices other than specialized resins/particles functionalized with divalent metal cations, such as bare iron oxide nanoparticles (K_D_ ~ 10^−6^–10^−7^ M) [32], [39]. In this context, the His6 tag has been successfully used to separate the target protein from bacterial cell lysates in a single-step purification process by binding to non-functionalized magnetic nanoparticles (Table 2) [32]. Immobilized Metal Affinity Chromatography (IMAC) matrices with enhanced selectivity to His-tag have also been developed by means of surface epitope molecular imprinting using this tag (Fig. 2) [40]. Additionally, the His-tag has been extensively used in several single-step protein purification and immobilization strategies that make use of its affinity toward surfaces functionalized with nickel (II) ions (Fig. 1A, Table 2) [29], [41], [42], [43], [44].

**Table 2:** Summary of different supports or matrices used in the last 5 years for tag-mediated protein purification and/or immobilization, with identification of corresponding application fields.

Type of support or matrix	Application field	Binding tag(s)	Strategy employed	References
**Metal/Metalloid-based materials**				
Bare iron oxide particles	Biotechnology and Biomedical	His6	Purification	[32]
Bare iron oxide particles	Biotechnology	Glu6	Purification	[45]
Iron oxide particles functionalized with amine groups	Biotechnology	Q6 (MTG-mediated)	Immobilization	[93]
Iron oxide particles functionalized with nickel (II) ions	Biotechnology	His6	Purification and Immobilization	[29]
Iron oxide particles functionalized with nickel (II) ions	Biotechnology	His6	Purification	[43]
Particles with an iron oxide core and a gold shell functionalized with nickel (II) ions	Biotechnology	His6	Purification	[44]
Gold surface functionalized with biotin protein ligase	Biomedical	Protein A (only the IgG-binding domain)	Immobilization	[124]
Silica particles	Biotechnology and Biomaterial	Car9	Purification and Immobilization	[50], [51], [52]
Silica particles	Biotechnology	SB7	Purification	[53]
Silica particles	Biotechnology	R5	Purification	[54]
Silica particles functionalized with amine groups	Biotechnology and Biomedical	Protein A (SrtA-mediated)	Purification and Immobilization	[91]
Particles with an iron oxide core and a silica shell	Biotechnology	clMagR and dMagR	Purification and Immobilization	[97]
Particles with an iron oxide core and a silica shell	Biomedical	pSN6	Immobilization	[57]
Particles with an iron oxide core and a silica shell functionalized with nickel (II) ions	Biotechnology	His6	Purification and Immobilization	[120]
Silica particles functionalized with 6-chlorohexanoic acid	Biotechnology and Biomedical	HaloTag®	Purification and Immobilization	[79]
**Carbon-based materials**				
Carbon nanotubes	Biomaterial	Lys6	Immobilization	[131]
Reduced graphene oxide	Biomaterial	Hydrophobin I	Immobilization	[8]
Graphene oxide particles functionalized with amine groups	Biotechnology and Biomedical	Protein A (SrtA-mediated)	Purification and Immobilization	[91]
**Polymer/Polysaccharides-based materials**				
SpyDock resin (iodoacetyl-activated crosslinked agarose beads functionalized with SpyDock)	Biotechnology and Biomedical	SpyTag or SpyTag002	Purification	[20], [38]
Sephadex® G-100 resin (crosslinked dextran) functionalized with SnoopCatcher	Biotechnology and Biomedical	SnoopTag	Purification and Immobilization	[72]
Crosslinked agarose functionalized with the affinity ligands A2C2 or A3C1	Biotechnology	Trp-based tags [(NW)3 or (WF)3]	Purification	[49]
Im7 resin (iodoacetyl-activated crosslinked agarose functionalized with Immunity protein 7)	Biomedical	CL7	Purification	[71]
Alfa-selector resin (crosslinked agarose beads functionalized with a single-domain antibody)	Biomedical	Alfa-tag	Purification	[76]
HaloLink™ resin (sepharose® beads functionalized with a chloroalkane)	Biotechnology and Biomedical	HaloTag®	Purification and Immobilization	[80]
Affi-Gel® 10 (N-hydroxysuccinimide-activated crosslinked agarose) functionalized with streptavidin (variant SAVSBPM32)	Biomedical	SBP-tag [A18C variant]	Purification and Immobilization	[73]
Sephadex® G-100 resin (crosslinked dextran) functionalized with SAVSBPM18-linker-DBD	Biotechnology	SBP-tag	Purification and Immobilization	[72]
Sepharose® resin (crosslinked agarose) functionalized with SAVSBPM18-linker-ABD(KC)	Biotechnology	SBP-tag	Purification and Immobilization	[23]
Superdex® 200 resin (cross-linked agarose and dextran)	Biotechnology	MhPA14	Purification	[70]
Sepharose® resin (crosslinked agarose) functionalized with nickel (II) ions	Biotechnology	(HE)7	Purification	[46]
Heparin Sepharose® resin	Biotechnology and Biomedical	HB	Purification	[74], [75]
Hyaluronic acid hydrogel functionalized with SpyTag-ELP-SpyTag	Biomedical	TriCatcher [SpyCatcher-ELP (with/without an RGDSP integrin-binding site)-SpyCatcher-SnoopCatcher]	Immobilization	[89]
Molecularly imprinted amine-based methacrylamide polymer MPS8	Biomedical	FLAG-tag® and DYKD	Purification	[77]
Cellulose	Biotechnology and Biomedical	CBMs	Purification and Immobilization	[65]
Cellulose	Biotechnology and Biomaterial	CBM64	Immobilization	[69]
Chitosan	Biotechnology and Biomaterial	CBM64	Immobilization	[69]
Starch	Biotechnology and Biomaterial	CBM64	Immobilization	[69]
Polypropylene	Biotechnology, Biomaterial and Biomedical	LCI	Immobilization	[132]
Acrylic-glass functionalized with nickel (II) ions	Biotechnology and Biomedical	His6	Immobilization	[41]
**Hybrid materials**				
Faujasite type alumino-silicate zeolite	Biotechnology and Bioremediation	pSN6	Immobilization	[56]
Particles with a silica core and a polyacrylamide shell functionalized with nickel (II) ions	Biotechnology, Biomaterial and Biomedical	His6	Purification and Immobilization	[40]
Particles with an iron oxide core and a PMG shell functionalized with nickel (II) ions	Biotechnology and Biomedical	His6	Purification and Immobilization	[42]
Glass coverslips coated with a gold film functionalized with nickel (II) ions	Biomedical and Bioengineering	His6	Immobilization	[127]
Reduced graphene oxide and nanofibrillated cellulose linked via a biocomplex	Biomaterial	hydrophobin I-RLP-(2)CBM	Immobilization	[60]

**Legend:** CBM, carbohydrate-binding module; DBD, dextran-binding domain; ELP, elastin-like polypeptide; LCI, liquid chromatography peak I (a polymer-binding peptide); MTG, microbial transglutaminase; PMG, poly(glycidylmethacrylate^–^methylmethacrylate); RLP, resilin-like polypeptide; SBP, streptavidin-binding peptide; SrtA, Sortase A.

Together with the His-tag, other peptides were recently reported to display affinity to iron oxide as well [39]. Among these, the Glu6 tag (Table 1) was also efficiently used in single-step affinity purification methodologies that employed non-functionalized magnetic nanoparticles as matrix (K_D_ ~ 10^−6^–10^−7^ M) (Table 2) [45]. In these studies, the strength of the interactions between these tags and the iron oxide nanoparticles could be modulated by varying the buffer conditions, namely composition, concentration and pH. Han et al. [46] demonstrated that a modified version of the His-tag, the (HE)7 tag (Table 1), fused to a truncated maltotriose-binding protein (MBP) exhibited higher yields of purified (HE)7-MBP-tagged protein when compared to His6-MBP-tagged protein, while also enhancing protein solubility. Another alternative to the His-tag recently developed was the 25-amino-acid NCTR25 tag (Table 1), truncated from the N-terminus of the human copper transporter 1 (hCtr1). Compared to His-tag, the NCTR25 tag was found to have less impact in the folding, kinetic and thermodynamic stability of the fused proteins while allowing similar purification efficiency by IMAC [47]. Thus, since both the (HE)7 and the NCTR25 tags can be used in place of the His-tag in standard IMAC protocols, these tags may be equally applied in single-step affinity purification and immobilization strategies.

Just like the His-tag, one of the earliest tags to be described was the cationic poly-arginine tag, which allows purification with cation exchange resin. Recently, new strategies have been developed to purify arginine-rich peptides, namely the Argi system based on the AR aptamer selected to capture Arg-based tags (K_D_ ~ 10^−7^ M) (Table 1 and Table 2) [48]. The properties of several tryptophan (Trp)-based tags have also been screened for single-step purification through the development of complementary affinity ligands [49]. Pina et al. [49] demonstrated that two new hexapeptide tag variants (Table 1) – (NW)3 and (WF)3 – fulfill the characteristics needed for a good affinity tag and showed that their system based in “tag-receptor” affinity pairs (K_D_ ~ 10^−7^ M) works similarly to other commonly used affinity-based purification systems. Therefore, these pairs can be potentially used for protein immobilization as well.

Other affinity tag-based purification systems have been developed with the aim of reducing the purification cost of recombinant proteins by replacing the ligand of the affinity columns (usually packed with metal ions) with more affordable natural materials. For instance, the 12 amino-acid Car9 tag (Table 1) was developed for single-step purification by exhibiting high-affinity binding to silica (K_D_ ~ 10^−6^ M) (Table 2) [50], [51]. According to the authors, the major advantages of this tag over other silica-binding tags (e.g., L2 and CotB1p tags) are its small size and needless of protease cleavage for target protein release [50]. This tag has been also used with success in microcontact printing methodologies, to direct the immobilization of proteins onto glass substrates (Table 2) [51], [52]. Similarly, Abdelhamid et al. [53] engineered a shorter version of the CotB1p tag – the SB7 tag comprising only 7-amino-acids (Table 1), which has also demonstrated high affinity to silica-based materials (K_D_ ~ 10^−9^ M). Like Car9, the SB7 tag uses an amino-acid buffer solution as a competitor eluent to dissociate tagged proteins from the silica materials (Table 2) [53]. Rapid and cost-effective recombinant protein purification methods that exploit the affinity of these tags to different low-cost silica matrices have shown to be comparable to or even better than the commonly used His-tag affinity purification system [50], [53].

The 19-amino-acid R5 bifunctional tag (Table 1), which displays silica-binding affinity (K_D_ ~ 10^−6^–10^−7^ M) and silica precipitation activity, has also been explored in single-step affinity purification (Table 2), but the purity levels achieved were unsatisfactory [54]. Another silica-binding peptide, the pSN6 tag (Table 1), which also displays affinity to some alumino-silicate zeolites (K_D_ ~ 10^−10^ M) [55], has been explored for protein immobilization (Table 2) [56], [57]. Although it has not yet been used for protein purification, likely due to it is large size compared to other silica-binding peptides with a more suitable size for such applications (Table 1), the affinity of pSN6 to different matrices makes it a particularly versatile tag to use in single-step protein purification and immobilization strategies.

**Fig. 3:**
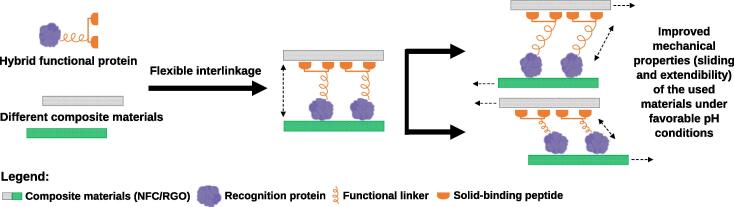
Multiple protein tag fusion used to generate bioinspired composites. Dhar et al. [60] constructed a multiple tag fusion where a resilin-like polypeptide (functional linker) was used as an elastic interface between two cellulose binding modules (a type of solid-binding peptide) and an amphiphilic hydrophobin (the recognition protein), thus forming a di-block functional protein with two recognition sites. This di-block functional protein was then used to act as a bridge to interconnect two layers of composite materials. While the CBMs specifically bind to nanocellulose (NFC) via site-specific recognition, the amphiphilic hydrophobin recognizes and adheres to reduced graphene oxide (RGO) via hydrophobic interactions, thus assembling a new structural and layered nanocomposite with enhanced stretchability due to the pH-dependent reversible conformational behavior of the functional linker. In the illustration, the direction of the applied tensile stress is represented by black arrows.

Another class of slightly larger affinity binding tags that in recent years has been mainly explored for protein immobilization are the carbohydrate-binding modules (CBMs) [58], [59], [60]. CBMs (Table 1), which were previously known as carbohydrate-binding domains, are protein domains derived from carbohydrate related enzymes that exhibit specific binding affinity to certain carbohydrate polymers. CBMs have been used for long as affinity tags for protein purification/immobilization of recombinant proteins [61] or even as a fusion partner for the recombinant production of antimicrobial peptides [62], [63], [64]. Also, CBMs have been fused with other peptides/proteins and applied in quite different contexts such as cosmetic [58], biosensor [65] or biomedical applications [62], [66]. CBMs have been also used in tandem, namely in multifunctional hybrid protein architectures such as the one recently used to develop a bio-inspired nacre-like nanocomposite (Table 2, Fig. 3) [60], or alone, to modify the properties of cellulose/cotton fibers [67], [68]. Meanwhile, recent insights on the CBMs’ structure and ligand plasticity have been reported [69]. Pires et al. [69] screened several CBMs and showed that the compact CBM64 variants (StCBM64B and StCBM64C with only 11 kDa) are versatile affinity tags with a high (K_D_ ~ 10^−7^ M) and extensive binding capacity to cellulose-based matrices under different and extreme physicochemical conditions. Therefore, these new CBMs hold the potential to outperform other CBMs currently used in protein immobilization strategies.

Other classes of larger affinity tags have been recently explored as well. Among these, the 22.5 kDa MhPA14-tag (Table 1) was reported to exhibit high affinity to dextran-based size-exclusion chromatography resins and was therefore tested as an affinity tag for single-step recombinant protein purification (Table 2) [70]. The newly developed dextran-affinity purification system outperformed the standard His-tag IMAC purification system [70]. Similarly, the ultra-high-affinity CL7/Im7 purification system (TriAltus, Biosciences) recently developed by Vassylyeva et al. [71] (Table 2) also presented clear superiority over the His-tag and other commercial affinity tag systems, allowing the obtainment of proteins that meet high-yield, -purity and -activity requirements [71]. This purification system is based on the protein/protein complex formed by Colicin E7 DNase (16 kDa) fused to the protein of interest (Table 1) and its inhibitor (Immunity Protein 7, 10 KDa), which displays an impressive binding affinity (K_D_ ~ 10^−14^-10^−17^ M) [71]. Thus, this ultra-high affinity system may be particularly useful for single-step protein purification and immobilization.

To prepare reusable affinity matrices suitable for the purification of biotinylated and streptavidin-binding peptide (SBP)-tagged proteins (Table 1), ~14 kDa truncated agarose- or dextran-binding domains (ABD, K_D_ ~ 10^−4^ M [23], or DBD, K_D_ ~ 10^−9^ M [72]) were recently used to mediate the immobilization onto Sephadex® of other peptides/proteins to which they were fused to, an engineered streptavidin (SAVSBPM18) (K_D_ toward SBP ~ 10^−8^–10^−9^ M) or the SnoopCatcher tag (Table 1) [23], [72]. The affinity matrices developed were successfully used for the original purpose, offering great flexibility for applications that require reversible or irreversible immobilization capability (Table 2) [23], [72], [73].

A novel rationally designed 32-amino-acid heparin-binding (HB) affinity tag (K_D_ ~ 10^−7^–10^−8^ M) (Table 1) was recently developed and used with success as an affinity tag in single-step purification of recombinant proteins [74], [75]. The use of polyclonal antibodies raised against the N-terminal of the HB tag exhibited high sensitivity in the detection of recombinant HB-fused recombinant proteins, thus further allowing the effective use of antibody-based separation methodologies [74].

Epitope-tagging is for long used in immunoaffinity-based protein purification/immobilization, being FLAG-, myc- and HA-tags the epitope tags most commonly used. Epitope-tagging, which still suffers from limited stability and strength of peptide-protein interactions, has been optimized. In this context, Götzke et al. [76] recently designed a novel ALFA-tag (NanoTag Biotechnologies) with only 1.7 kDa (Table 1) for protein detection and purification that seems to address many limitations of conventional epitope tags, mainly those related with immunofluorescence [76]. In fact, this small and highly versatile epitope tag shows strong affinity (K_D_ ~ 10^−8^–10^−11^ M) to Alfa-selector resins (NanoTag Biotechnologies) and is compatible with standard fixation methods, thus being suitable for the detection of ALFA-tagged proteins by either conventional or advanced microscopy [76]. Additionally, this technology allows highly efficient nanobody-based single-step protein purification [76]. This engineered ALFA-tag appears as a promising new tool for biomedical and biopharmaceutical industries.

To overcome the use of highly expensive antibody-based resins, the FLAG®-tag (Sigma-Aldrich) (Table 1) and the tetrapeptide DYKD contained in the FLAG epitope have been recently used as templates to develop molecularly imprinted polymers (MIPs) with high affinity to these tags (Fig. 2). The imprinted resins were successfully used to purify FLAG- and DYKD-tagged proteins from crude biological extracts (Table 2) [77]. This new purification system outperformed that based on Immunoaffinity columns in terms of purity, recovery yields and cost-effectiveness [77].

### Covalently binding tags

Covalently binding tags allow the rapid, highly specific and irreversible binding of recombinantly tagged proteins to specific ligands (Fig. 1B). In turn, those ligands may be covalently/non-covalently attached to other molecules (e.g., labels) or materials. Covalent immobilization tag systems such as the widely known HaloTag® (Promega), SNAP-tag® (New England Biolabs) and CLIP-tag® (New England Biolabs) are based on enzymes that catalyze their covalent attachment to specific synthetic ligands [78], [79]. Among these, the HaloTag® (Table 1) is the most used for protein immobilization, also allowing the isolation and purification of proteins at levels unachievable by traditional protein affinity purification methods [78]. This system is based on a modified haloalkane dehalogenase that specifically recognizes terminal chloroalkane ligands, with which it establishes a stable covalent ester bond via a self-terminating mechanism [78], [80]. In 2017, Döbber and Pohl [80] developed a rapid, selective and stable site-specific covalent binding method based on an engineered version of the original HaloTag®, which displays improved soluble production. They demonstrated that this engineered tag allows the covalent immobilization and purification of proteins from crude biological extracts in a single-step, being suitable for enzyme immobilization [80], [81], [82].

**Fig. 4:**
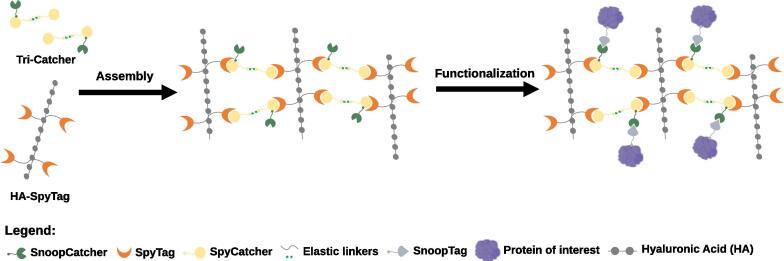
Exploration of the Tag/Catcher system properties for the self-assembly and functionalization of engineered protein-based hydrogels. By coupling HA-SpyTag (HA coupled to two SpyTags linked by an elastin-like polypeptide) with TriCatcher (two SpyCatcher linked by an elastin-like polypeptide with/without an RGDSP integrin-binding site and a C-terminal SnoopCatcher), Wieduwild and Howarth [89] rapidly assembled stable hyaluronan hydrogels via SpyTag/SpyCatcher isopeptide bond formation. Functionalization was further achieved by linking SnoopTagged-proteins to the SnoopCatcher without interfering with the hydrogels’ mechanical behavior.

During the last decade, a novel and promising approach based on irreversible conjugation of recombinant proteins was developed [83]. This emergent approach was based on the rational splitting of bacterial domains containing intramolecular isopeptide bonds into two parts, a Tag peptide and a Catcher protein (Table 1) [83], [84], [85]. When the Tag becomes in contact with its corresponding Catcher, the interrupted intramolecular isopeptide bond is spontaneously reconstituted, rapidly forming a stable covalent bond [37], [38]. Therefore, the Tag/Catcher system enables the rapid and irreversible linking of proteins that are fused to the Tag or the Catcher, eliminating the need of chemical modification, artificial amino acids or cysteines for covalent bond formation [83], [84], [85]. The Tag/Catcher system thus allows strong linkage via a covalent bond, offering a unique protein architecture adaptable to a wide range of applications, namely one-step purification and immobilization of proteins [37], [85], [86], [87]. The SpyTag/SpyCatcher pair was the first system pair engineered by Howarth’s lab [83] and further improved by the same research group in order to achieve faster reaction rates (SpyTag002/3:SpyCatcher002/3) when compared to the original pair [20], [88]. Further engineering of the SpyCatcher led to the generation of an unreactive SpyCatcher (K_D_ ~ 10^−7^–10^−8^ M), based on which a new affinity matrix (named SpyDock) was developed for the purification of SpyTag fused proteins (Table 2) [38]. Other Tag/Catcher pairs were further developed by finding orthogonal proteins from different bacterial species, such as the SnoopTag/SnoopCatcher [84] and the SdyTag/SdyCatcher [36], and used for protein assembly and construction of novel protein architectures. One of such architectures involves the use of the SpyCatcher and SnoopCatcher in tandem in the TriCatcher protein, which allowed assembling and decorating hyaluronan hydrogels (Fig. 4) [89]. The tags used in the Tag/Catcher systems are small (<2 kDa) and the isopeptide bond formation is fast, precise and permanent under a diverse range of conditions [38], [83], [84], offering a major advantage when compared to other covalent coupling tags not suitable for direct interaction with other proteins.

In addition to self-ligating systems, chemoenzymatic approaches have also been explored for single-step purification and covalent immobilization of proteins on solid supports [90]. In this context, the transpeptidase Sortase A (SrtA), which canonically recognizes substrates with the LPXTG sorting signal, was recently used to mediate the single-step purification and oriented immobilization of protein A (which naturally has the LPXTG motif) onto silica and graphene oxide nanoparticles functionalized with nucleophile donor groups (Table 2) [91]. In the presence of calcium, SrtA forms a thioester intermediate by cleaving the amide bond between the threonine and glycine in the LPXTG motif, which is then displaced by an amine nucleophile, typically the free amino group of a polyglycine substrate, forming a stable isopeptide bond and allowing the recycling of the enzyme [92]. The functionalized nanoparticles were further used for the purification of Human Immunoglobulin G, thus confirming that the immobilized protein A retained its functional properties (Table 1) [91].

Microbial transglutaminases (MTGs), which catalyze the formation of a covalent bond between a free amine group and the side chain of a glutamine residue on one protein or peptide, have also been used to mediate the oriented and covalent immobilization of Q6-tagged enzymes onto solid substrates/supports (Table 2) [93]. New highly reactive Q-tags for the *Bacillus subtilis* MTG (Table 1) [94] and new MTGs with high substrate specificity [95] have been recently reported, providing new tools for MTG-mediated covalent immobilization of recombinant proteins, biomaterial formation and site-specific protein modification.

### Alternative purification tags

As mentioned in the previous subsections, covalently and non-covalently binding tags are appended to recombinant proteins so that they can be purified from their crude biological extracts using a wide range of chromatographic techniques. Although efficient, these tags usually bind to specific matrices, thus demanding specialized resources. In the last years, alternative systems have been developed to simplify the purification process, following an economic and ecological perspective [96], [97]. For instance, non-chromatographic and matrix-free purification systems using self-aggregating tags that undergo a phase transition from soluble to insoluble have emerged in the last years and were reviewed in detail elsewhere [96], [98]. Those self-aggregating tags induce the formation of aggregates when fused to the protein of interest, which can be then separated from crude biological extracts upon changes in the temperature, pH or ionic conditions [98]. A self-cleaving protein or a protease recognition site is usually cloned between the tag and the protein of interest to allow tag release. This purification system is known to be more cost-effective and easier to scale up than conventional chromatographic techniques and has been broadly explored for biotechnology, biomedical and pharmaceutical applications (Fig. 1C) [98].

The elastin-like polypeptide (ELP) tag (Table 1) is an example of a self-aggregating tag intensively explored for biotechnological and biomedical applications, mainly for the functionalization of polymer-based materials [99], [100] and for long used under a variety of ways to purify proteins [101], [102]. It is a natural polymer that under certain temperatures and salt concentrations reversibly forms precipitates with the fused protein, facilitating the purification of the protein of interest, which is then recovered from the crude extracts via centrifugation. In these strategies, the use of tandem repeats of the affinity ligand can improve the capture efficiency [103]. The versatility of this approach has also been shown with the successful purification of different proteins [103], [104] and it may also be considered for immobilization purposes if multiple affinity ligands are used.

The pH-responsive CspB50 tag (5.4 kDa; Table 1) is another example of a self-aggregating tag. Proteins fused with the CspB50 tag were shown to reversibly precipitate at acidic pH, thus being purified using a column-free method with yields and purity comparable to those obtained with conventional affinity purification systems. According to the authors, advantages of the CspB50 tag over ELP tags are: (i) the aggregating-state is fully reversible, (ii) it has a sharp pH responsivity, and (iii) it can have multipurpose applications [105], [106].

Both the thermo-responsive ELP and pH-responsive CspB50 reveal to be very attractive tags for non-chromatography-based protein purification methods, being emergent alternatives to conventional matrices [101], [105]. However, most of the interactions of self-aggregating tags rely on environmental conditions such as temperature, pH and ionic strength, which may not be suited to maintain the activity of the tagged protein [103], [104]. To overcome these limitations, other types of tags have been recently explored, namely for magnetic-absorption-based purification and immobilization of proteins [97]. These tags are based on a magnetoreceptor protein – MagR (~14.5 kDa; Table 1) and employ a magnetic-based approach to easily recover the protein of interest form crude biologic extracts in a single step. Briefly, the protein of interest fused with the MagR tag is absorbed to magnetic beads, followed by aggregation and purification/immobilization [97]. Qin et al. [107] reported the first MagR (from *Drosophila melanogaster*) in 2015 and later, Jiang et al. [97] used this (renamed as dMagR) and other MagR from *Columba livia* (clMagR) as tags for the recycling of immobilized enzymes with improved thermostability and enhanced pH tolerance when compared to the free enzyme. The magnetic interactions between the MagR tag variants and the recovery support were reported to be less affected by the chemical environments [97]. Similarly, this magnetic interaction was successfully used to purify other enzymes in a single step [108]. Both studies have shown that different enzymes can be immobilized over a broad range of environmental conditions by using the MagR tag variants without compromising enzyme activity.

## Tag-mediated purification and immobilization of proteins: Recent advances and applications

Besides its central role in purification processes, tags are vital when the aim is to have the peptide/protein of interest immobilized onto a specific matrix (usually used to enhance peptide/protein stability and to preserve their biological activity) [23], [30] or in an engineered material (functionalized with one or more biologically active peptides/proteins of interest) [2], [35], [60]. In these cases, single-step purification and immobilization methods based on the use of tags that specifically link the peptides/proteins of interest to the surface of organic or inorganic materials have open the way for an array of diverse novel protein/peptide applications. In this context, protein purification tags such as those mentioned in Section ‘Protein purification/immobilization tags’ are pivotal and show great potential for anchor peptide/protein-based technology. They are used alone, or conjugated with other peptides or proteins, as covalent or non-covalent linkers for material surface functionalization without affecting the properties of the materials or interfering with the fusion partner. Recent developments within this context and in the context of novel purification processes are explored in this section.

In the scope of His-tag purification processes, creative approaches are currently being explored by expanding and adapting the IMAC principle to achieve a faster and more efficient purification process without the need for an expensive and laborious column system. One of those innovative systems rely on the use of nanoparticles, mainly magnetic nanoparticles (MNPs) composed of magnetic elements (e.g., iron, nickel or cobalt) and their oxides (e.g., magnetite, maghemite or cobalt ferrite), and a tag attached to the protein of interest that interacts with the MNPs' surface (coated or not) through reversible interactions [39], [97], [108], [109], [110], [111]. MNPs encompass several advantages, such as easy manufacture and manipulation, small size, stability and reusability. Nanoparticles with or without a magnetic core usually show good biocompatibility (e.g., non-immunogenicity and resistance to plasma protein opsonization) and good biodegradation properties, nevertheless these properties often depend on the synthesis method employed [110], [111], [112]. Due to their unique physicochemical properties and versatility, MNPs have potential roles in a number of scientific and technological areas, including in biomedical (e.g., disease diagnosis and therapy), bioengineering (e.g., biosensor and bioelectrode), bioremediation (e.g., water treatment) and biotechnological (e.g., material functionalization and biomolecule immobilization and purification) applications [110], [112]. Protein purification mediated by MNPs usually encompasses the following steps (Fig. 1A): (i) specific adsorption of the tagged protein onto the MNPs in a buffer-controlled reversible interaction and its magnetic separation from impurities, (ii) desorption (or elution) of the tagged protein from the MNPs by changing the buffer composition (similarly to what is done in IMAC purification), and finally (iii) isolation of the tagged protein of interest from the MNPs using an external magnetic field. This magnetic adsorption-based separation method can be used in any downstream process and eliminates steps during the purification, offering an easily automated and scalable alternative to the conventional methods [112]. Despite the simplicity of the process, it is known that the interactions of the tag-protein fusion with the MNPs’ surfaces are complex and highly dependent of the environmental conditions and physicochemical properties of the tag-protein fusion, such as surface energy, polarity, morphology and charge [39], [113].

MNPs can be used uncoated or coated with functional groups or complex ligands, or even embedded in polymeric matrices to provide different specificities (Table 2) [113], [114], [115]. Although the manufacturing process of bare iron oxide MNPs is simple and does not require the use of aggressive or hazardous agents (e.g., toxic metal ions), coated MNPs are less exposed to degradation, displaying better stability and less tendency to aggregate [115], [116]. For most *in vivo* applications, and despite some limitations such as increased manufacturing costs and some ecological and public health reservations, MNPs’ surface needs to be coated [115], [116], [117], [118]. Although less used, bare iron oxide (uncoated) MNPs have already been successfully employed to purify His6- and Glu6-tagged recombinant proteins from crude biological extracts in a single step [32], [45]. Uncoated MNPs' tendency to aggregate lowers the effective surface area for protein/peptide immobilization, influences the MNPs properties (e.g., binding behavior, separation performance and long-term stability of the recovered product), and also increases the predisposition to a faster clearance from the human body mediated by phagocytes. Coating the MNPs leads to a reduction of these side effects by stabilizing the surface of the nanoparticles, minimizing aggregation and agglomeration, while enhancing the targeting function and ensuring a nontoxic status in physiological conditions [115]. MNPs can be coated with materials from either synthetic or natural sources (Table 2), thus forming a magnetic composite at nano or micro scale. The functionalization of the surface of iron oxide MNPs with immobilized nickel (II) ions (Ni^2+^) – an IMAC adaptation – is commonly used to achieve rapid and efficient purification (in terms of binding rate) of His-tagged proteins and also to immobilize a variety of biomolecules in a fast and cost-effective way (Table 2) [31], [42], [119]. Similarly, silica-coated MNPs functionalized with nickel (II) ions [120] or modified with functional groups such as amine [93] or even with thiol [121] groups, are also commonly used for enzyme immobilization (Table 2). Additionally, silica and graphene-based nanoparticles without a magnetic core and functionalized with nickel (II) ions [40] or free amine group [91] were also successfully employed to enhance immunoaffinity-based detection and purification, and to immobilize proteins without activity loss, thus demonstrating the wide applicability range of nanoparticles [24], [122].

The functionalization of materials’ surface for improving their functional and/or structural properties has indeed gained a stereoscope application in research and industrial bioprocesses. Natural and traditionally used polymers and polymer-based or conjugated materials have been functionalized with affinity ligands [70], [72], [73], aptamers [48] or antibodies [71], [123], [124], [125], [126] by making use of novel engineered tags as fusion partners (outlined in Table 2). In comparison to random immobilization, site-directed (oriented) immobilization of antibodies onto solid surfaces (via physical adsorption or cross-linking) shows superior performance, by enhancing antibody-binding capability, which results in signal amplification and thus in highly sensitive immunoassays [21].

Materials such as glass and acrylic glass functionalized with nickel (II) ions have also been employed in affinity-mediated oriented immobilization of His-tagged recombinant proteins [41], [127]. However, despite the higher cost and limited availability, inert metals such as gold are still preferable for biomolecule-oriented immobilization due to their stability, biocompatibility, ability for bioconjugation and enhanced physicochemical properties (e.g., optical, electronic and catalytic) [124], [128], [129], [130]. Additionally, functionalized materials within a single-step such as carbon-based [8], [60], [131] and polymer-based matrices [60], [65], [67], [133] are also successfully used in several biomedical applications ranging from regenerative therapy to smart sensors made from natural or synthetic fibers.

Another field in exponential growth is the creation of novel matrices for specific protein purification and/or immobilization mainly for biomedical use (see polymer-based materials section in Table 2). For instance, synthetic materials such as MIPs have recently emerged as artificial but effective substitutes for the specific recognition of biological elements such as purification tags, antibodies and enzymes (Fig. 2) [40], [77], [125], [134], [135]. Comparatively to other matrices, MIPs have the advantage of being more stable and robust, less expensive and eventually reusable, appearing as a viable alternative to well-established IMAC and antibody-based resins for single-step purification of recombinant proteins in terms of selectivity, affinity and binding capacity [40], [77], [125], [134], [135], [136]. MIPs have been mainly used in the form of beads packed in affinity purification columns [40], [77], but cellulose membrane surfaces modified with epitope- or protein-imprinted polymer hydrogel films have been also used as affinity membranes for the purification of recombinant antibodies [125]. An important advantage of macroporous membranes over packed-bed columns is that their binding capacity is independent of the flow rate over a wide range of values, which allows them to work at higher flow rates, reducing process time and buffer consumption [125]. Protein-engineered matrices functionalized with different tags – initially designed for purification, have also been developed and seem to display enhanced flexibility for applications that require reversible and irreversible immobilization capacity [72], [73]. On the other hand, innovative polymer-based matrices based on a mix-and-match approach have been created through transglutaminase-mediated crosslinking for the immobilization of Human growth factors [133], via oriented and highly-selective conjugation for the purification/immobilization of ALFA-tagged recombinant proteins (the nanobody-based ALFA Selector resin) [76], or mediated by the self-ligand properties of the Tag/Catcher system, which prompts the self-assembly of novel matrices for biomedical applications (Fig. 4) [89], [137]. These self-assembled protein-based hydrogels were rapidly formed and showed superior adhesion and absorption/desorption properties [89]. The Tag/Catcher technology ultimately enables the production of tailored molecular networks at very high densities via site-specific bioconjugation of the Catcher partner to different polymeric materials [38], [72], [89]. Thus, this technology could be further extended to other substrates or peptide/proteins pairs, potentially generating novel and advanced engineered biomaterials with enhanced functionality and/or tailored for specific applications [37], [137], [138], [139], [140].

The exploitation of functionalized protein-based materials such as silk, collagen, elastin and genetically engineered recombinant protein polymers, and their use in a myriad of biomedical, bioengineering and textile applications, ranging from scaffolds for tissue engineering to smart and functional textiles, has gained a new impetus, going beyond the development of novel matrices for protein purification [141], [142], [143], [144], [145]. This was due to the progress in biomedicine and nano-fabrication technologies, and to their excellent biocompatibility and unique mechanical properties, [141], [142], [143], [144], [145]. For instance, the creation of hybrid composites combining at least two different materials at nano or molecular scale (recent developments outlined in hybrid materials section in Table 2) has been broadly utilized in many technological, biological and structural applications, ranging from the creation of textiles with enhanced properties to soil and water bioremediation [145], [146], [147], [148], [149], [150], [151]. These hybrid materials display superior and tunable properties, as synergetic characteristics consistently arise from materials’ interaction [5], [152], [153].

Innovative multifunctional materials made from functional recombinant proteins that self-assemble in a way that enhances their function and simplifies the immobilization/purification process, making use of more cost-effective and sustainable technologies, is another field in exponential growth. Reviews specifically concerning this topic are available elsewhere [153], [154]. Such highly dynamical and spatially structured assembled materials are now broadly used in a range of applications such as those requiring a stimuli-responsive function (Fig. 3) [1], [141], [145], [146]. In this field, CBM-tagged proteins grabbed particular interest as substitutes of the traditional reinforcements made of glass or carbon fibers, due to their high specificity and sensitivity for the different crystalline faces of cellulose and to their exceptional mechanical properties for effective single-step purification and immobilization [33], [60], [69], [155]. For specific applications, protein-based materials are somewhat preferable due to their enhanced multifunctional properties such as flexibility, biodegradability, and extraordinary adsorption affinity over other composites. Additional properties such as stability, toxicology and biocompatibility also give them potential in the fields of biomedical engineering [156], [157], [158], [159].

Although there has been significant progress, the immobilization of peptides/proteins onto supports is not a straightforward process, involving highly complex molecule-material interactions that are still not fully understood. Fundamental and technical knowledge needs to be ascertained, including: (i) strength and stability of the interaction between the peptides/proteins and the supports under relevant processing conditions; (ii) influence of the materials’ surface *per se* in the final assembly; (iii) retention of the materials’ properties after the process; (iv) and very important, how to control the physico-chemical factors that lead to the attachment of peptide/proteins to the surface of the material in a manner that preserves their conformation and biological activity [22], [160], [161], [162]. Despite the technological advances made, many studies fail to report the binding kinetics/thermodynamics of the molecule-material complex, which hampers the understanding of fundamental energetic insights of a series of molecule-material interactions [163]. For instance, only a third of the studies mentioned in table 2 have quantified the binding affinities of the indicated molecule-material complex. Among these, the binding affinities were reported either in the form of Gibbs free energy or association/dissociation equilibrium/rate constants. Simplification, harmonization and standardization of the methodologies and representations used would provide a more straightforward comparison between studies. Within the scope of single-step protein purification and immobilization, the loading capacity of the matrices, as well as the specificity and stability of the interactions between the tagged proteins/peptides and their corresponding matrices will continue to pose obstacles to the efficiency of such strategies, which will only be overcome with a more fundamental knowledge of such interfacial interactions.

## Summary and future outlook

The emerged possibilities of protein-engineered materials grabbed the attention of researchers and stakeholders and are now considered the future of material science. Within this context, the sustainable supply of proteins/peptides required for the development of such materials relies on recombinant protein production and on purification and immobilization technologies that have significantly evolved over the last years. Tag-mediated protein purification/immobilization strategies have emerged as eco-friendly and cost-effective alternatives to conventional chemical immobilization methods, allowing the combined purification and immobilization of proteins/peptides onto natural, synthetic or hybrid materials in a single step, and ultimately, the simplification of the fabrication process of protein-engineered materials. Among the tags summarized in this review, covalently binding tags appear as most suitable for long-term immobilization of proteins but lack versatility in what concerns the materials to which they can bind to without the need of any chemical modification (only protein-based materials). Conversely, affinity tags have the ability to bind to a wide range of materials (of synthetic, natural or hybrid nature), being more suitable for protein purification than for long-term protein immobilization. Therefore, several strategies have explored the used of hybrid affinity-covalently binding tags to allow the efficient one-step purification and/or immobilization of proteins onto different materials, as well as the development of innovative protein-engineered materials. Within this context, self-aggregating responsive tags have also been particularly useful in combination with other tags for the development of protein-engineered materials with self-assembling, flexible and/or responsive properties.

The orthogonality and versatility of SBPs has undoubtedly pinpointed the exploitation of functional proteins for biomaterials, potentiating novel perspectives toward new bioprocesses and advanced materials with new/improved biological and/or structural properties useful for applications in several industrial sectors, namely in the traditional textile and electronic sectors. Recently, a new generation of covalently binding tags, such as those within the Tag/Catcher system, boosted the design of more stable and multifunctional protein architectures adaptable to a wide range of applications. Despite the advances made, the design, engineering and control of protein-material interfaces at the molecular level will continue to face some drawbacks. Ongoing novel, creative and sustained developments and approaches will be required to tackle these new challenges. Still, the potentialities of protein-engineered materials are so wide and exciting that many advances are expected in the forthcoming years within this area of research. We have recently witnessed the creation of: (i) bio-inspired nacre-like composites made of graphene and nanocellulose “glued” via a multiple tag fusion [60], (ii) novel catalytic nanoreactors, vaccine delivery platforms and coatings based on self-assembled protein cages loaded/decorated with different proteins via Tag/Cather conjugation [164], [165], [166], (iii) and of electrically conductive protein nanowires functionalized with SBPs that show expanded properties useful for the development of eco-friendly electronic materials [167]. Therefore, the future of this field will only be limited by our imagination.

To guarantee the economic and ecological sustainability of these new materials with new or enhanced functionalities, novel and highly efficient single-step protein purification and immobilization strategies are envisioned to appear in the imminent years, namely boosted by a deeper understanding of the interfacial interactions between materials’ surfaces and tagged proteins/peptides. The insights provided in the present review are intended to guide, motivate and inspire breakthroughs in this area of research.

## Compliance with Ethics Requirements

This article does not contain any studies with human or animal subjects.

## CRediT authorship contribution statement

**Ana I. Freitas:** Conceptualization, Investigation, Visualization, Writing - original draft. **Lucília Domingues:** Conceptualization, Supervision, Writing - review & editing. **Tatiana Q. Aguiar:** Conceptualization, Supervision, Validation, Writing - review & editing, Funding acquisition.

## Declaration of Competing Interest

The authors declare that they have no known competing financial interests or personal relationships that could have appeared to influence the work reported in this paper.
